# Identification of fetal cardiac anatomy and hemodynamics: a novel enhanced screening protocol

**DOI:** 10.1186/s12884-016-0933-9

**Published:** 2016-06-30

**Authors:** Ying Zhang, Ai-Lu Cai, Wei-dong Ren, Ya-Jun Guo, Dong-yu Zhang, Wei Sun, Yu Wang, Lei Wang, Yue Qin, Li-ping Huang

**Affiliations:** Department of Sonography, Shengjing Hospital of China Medical University, No. 36 Sanhao Street, Heping District, Shenyang, Liaoning China

**Keywords:** Prenatal diagnosis, Cardiac screening, Congenital heart disease, Fetal echocardiography, Segmental analysis

## Abstract

**Background:**

Prenatal cardiac screening is of great importance as it contributes to appropriate neonatal management and helps parents to make a decision regarding their pregnancy. The aim of our study was to evaluate the efficiency of a newly proposed screening protocol in the detection of fetal congenital heart disease (CHD).

**Methods:**

This was a prospective study. A total of 52 cases of confirmed CHD fetuses and 248 cases of randomly selected normal fetuses were included in the study. Two sonographers with similar experience performed the cardiac screenings under two different protocols independently. The conventional protocol (Protocol A) paid greater attention to the four-chamber view and the outflow tract views. A 6-month training program was provided to sonographers performing scans under the new protocol (Protocol B), which emphasized systematically evaluating fetal cardiac anatomy and hemodynamics. Color Doppler was mandatory and some ultrasonic signs for special cardiac anomalies were also introduced into this protocol.

**Results:**

Protocol B detected more cardiac anomalies than did Protocol A (96.2 % vs. 61.5 %, *P* < 0.01). Specifically, Protocol B was superior to Protocol A in detecting cardiac malpositions, abnormal systemic and pulmonary venous connection, right aortic arch, transposition of the great arteries, and congenital corrected transposition of the great arteries. By visualizing flow disturbance and retrograde flow with color Doppler, Protocol B was better than Protocol A in screening valvular associated malformations, such as pulmonary atresia, pulmonary stenosis, tricuspid dysplasia, etc. For the normal fetuses, Protocol B was better than Protocol A in reducing the false-positive detection of septal defects.

**Conclusions:**

The current study introduces an enhanced protocol for fetal cardiac screening, under which the obstetric screening sonographers systematically identify fetal cardiac anatomy and hemodynamics. A short-term training program makes it possible for the screening sonographers to become familiar with the new protocol, and its value has been confirmed due to improvements made in screening efficiency.

**Electronic supplementary material:**

The online version of this article (doi:10.1186/s12884-016-0933-9) contains supplementary material, which is available to authorized users.

## Background

Congenital heart disease (CHD), accounting for about 0.4–1.3 % of all live births [1–4], is the most common congenital malformation leading to perinatal morbidity and mortality, and is considered the leading cause of death in newborns with congenital anomalies [5, 6]. Fetal echocardiography is undoubtedly the best method currently available to diagnose congenital cardiac anomalies prenatally [7]. However, it is not practical for fetal echocardiographers to make a diagnosis for every fetus during routine obstetric scans. Thus a detailed cardiac screening performed by obstetric sonographers may be an alternative to solving the problem [8–10]. When CHD is suspected, patients should be referred for comprehensive cardiac examination by a fetal echocardiographer. To this end, highly efficient screening might then lead to a high detection rate for fetal CHD.

A symmetric four-chamber view (4CV) has been used to confirm a normal fetal heart since the early 1990s [11, 12]. However, it is well-documented that 4CV alone is inadequate in ruling out conotruncal anomalies [13–15]. The ISUOG guideline [16] has been revised and updated to include more cardiac views that improve the efficiency of prenatal screening for CHD. In our center, 4CV, combined with double outflow tract views and three-vessel and trachea view (3VT), has been used in routine obstetric screening since 2005. It is confirmed that this produces more effective referrals than ever before. However, we found that there were still many serious CHDs that were missed during screening. We therefore propose an improved screening protocol to enhance current cardiac screening methods.

## Methods

### Study population

In total, 253 randomly selected pregnant women who signed their informed consent, and would undergo two screening examinations were included in the current study between Oct 2012 and Oct 2014. As this study was designed to evaluate efficiency of the newly proposed protocol for cardiac screening, we decided to include as many CHD fetuses as possible to strengthen our study, although it was impossible to acquire all the CHD cases during routine obstetric screenings. Pregnant women (a total of 47 cases) whose fetuses had been diagnosed with fetal structural cardiac anomalies in the fetal echocardiography center of our hospital and signed informed consent were also invited to participate in this program between Oct 2012 and Oct 2014.

These patients then underwent thorough screening examinations (including cardiac screening), performed by two obstetric screening sonographers with 2 years of experience independently. Sonographer A performed the scans and made immediate diagnosis under the current screening guidelines (Protocol A). Sonographer B, which was trained for 6 months to become familiar with the proposed protocol (Protocol B), performed the same work under Protocol B.

All fetuses involved in the program were from singleton pregnancies, and the gestational age at scanning ranged from 20 to 24 weeks (mean, 22.4). The screening sonographers involved in the program were not aware of the purpose of the study; and they were also blinded to the patients’ information and prior fetal echocardiographic imaging reports. Three Doppler ultrasound systems (Voluson E8, GE Healthcare, Kretztechnik, Zipf, Austria), each equipped with a 4–8 MHz and a 2–5 MHz transabdominal transducer, were used in our study. All data were saved as video clips. The time for cardiac scans and image interpretation under each protocol was recorded.

### Ultrasonography technique

#### Protocol A: transverse sweep from 4CV to 3VT

This protocol was mainly consistent with the one recommended by ISUOG [16]. Briefly, a clear 4CV was acquired with apical or lateral insonation of the fetal heart. The symmetry of the four chambers, atrioventricular coordination, the position where the atrioventricular valves insert into the septum, the separate and free opening of these valves, and the continuity of cardiac crux and interventricular septum were evaluated in 4CV. On the basis of 4CV, the ultrasound beam was gradually turned up to the fetal head to acquire the left and right outflow tract view and the 3VT, through which anomalies associated with conotruncal could be detected. In this protocol, the use of color Doppler was not considered mandatory.

#### Protocol B: systematic study of fetal cardiac anatomy and hemodynamics, with awareness of some ultrasonic signs of specific anomalies

Sequential segmental analysis was systematically used in this protocol to identify the fetal visceral and cardiac position, and morphologic structures at the atrial, ventricle and arterial levels in an orderly and sequential fashion.

The first step was to determine the fetal visceral and cardiac position, which had been described in detail in our previous report [17]. Briefly, a long-axis plane of the fetus combined with the transverse planes at both the fetal abdominal level and thoracic level were used to ascertain whether both the fetal stomach and heart were on the left side of the fetus.

Continuous transverse scans from the 4CV level up to the left/right outflow tract level, and up to the 3VT level were then performed for each fetus. In addition, several points were emphasized in this protocol:

1. Some important anatomical structures are meant to be identified, such as pulmonary veins (PV) (at least one left PV and one right PV), superior vena cava (SVC), inferior vena cava (IVC), left/right atrium (LA/RA), left/right ventricle (LV/RV), atrioventricular/semilunar valves, foramen ovale, ovale valve, aorta (ascending aorta and the arch), pulmonary artery (PA) (main PA, bifurcation, and the left and right PA) and the ductus arteriosus (DA).
2. The connection of the two adjacent segments is to be affirmed clearly; for example, PV connected with LA, LA connected with LV, LV connected with aorta, and the confluence of ductal and aortic arches. To visualize certain anatomical structure, the scans should not be limited to acquiring the transverse view. For example, when 4CV appeared, we rotated the ultrasound beam to the long-axis plane along the RA to confirm that IVC and SVC were connected with RA.
3. Color Doppler was very important as it could identify abnormal intracardiac blood flow patterns such as signals across two ventricles, suggesting the existence of a ventricular septal defect (VSD). Color Doppler also made it easier to disclose valvular structural abnormalities compared with 2D sonography (Table 1). In addition, it identified whether the flow direction was correct at certain anatomical locations and determined the existence of abnormalities by analyzing cardiac hemodynamics (Table 1).

**Table 1:** Indications of color Doppler findings in fetal cardiac screening

Abnormal blood flow patterns	Indications
Suggest vavular structural anomalies	
High velocity blood flow detected at atrioventricular/semilunar valves	Valvular stenosis
Severe TR but with low velocity	Tricuspid valve dysplasia or Ebstein’s anomaly
Non-restrictive pulmonary regurgitation, combined with dilated MPA, LPA, and RPA	Absent pulmonary valve syndrome
Suggest an indirect sign	
MR with high velocity	Aortic valve stenosis/atresia
TR with high velocity	Pulmonary valve stenosis/atresia; premature closure of the ductus
Antegrade flow in the DA and retrograde flow in the aorta at 3VT	Aortic valve atresia or COA
Antegrade flow in the aorta and retrograde flow in the DA at 3VT	Pulmonary valve stenosis/atresia

*3VT* three-vessel-trachea view, *COA* coarctation of aorta, *DA* ductus arteriosus, *MR* mitral regurgitation, *LPA* left pulmonary artery, *PA* pulmonary artery, *PR* pulmonary regurgitation, *RPA* right pulmonary artery, *TR* tricuspid regurgitation
4. When an abnormal structure is suspected, continuous scans around the anomaly should be performed to determine its source and propose a putative diagnosis.

To summarize, this protocol emphasized the detection of cardiac anomalies on the basis of a good understanding of normal cardiac anatomy and hemodynamics. Understanding the anatomy and pathophysiologic changes in various CHDs was also encouraged. This protocol also allowed recognition of specific ultrasonic signs of certain cardiac anomalies from various visual perspectives (Table 2); some of these reference Jeanty et al. [18–21].

**Table 2:** Abnormal structures that are important for cardiac screening sonographers

Abnormal signs	Possible anomalies
Up-abdominal and 4CV level	
Associated with cardiac malposition	
The heart and the gastric vacuole are both on the right side	Mirror-image heart or complete situs inversus
The heart is on the right side while the gastric vacuole on the left side	Dextrocardia; suggesting a high incidence of CHD
The heart is on the left side while the gastric vacuole on the right side	Heterotaxy; suggesting a high incidence of CHD
4CV shows the displacement of the heart to the right side	Cardiomediastinal shift caused by extra-cardiac situations
Associated with systemic/pulmonary veins connection	
A vein flowing cranially was visualized adjacent to the DAO at the 4CV	It may be the azygos or hemizygous continuation of IVC interruption. It is necessary to determine whether IVC is connected with RA
A vein flowing caudally was visualized next to the DAO at the 4CV	It may be TAPVC with an infradiaphragmatic connection. It is necessary to confirm whether PVs is connected with LA.
Vein-like structure was visualized between DAO and LA at the 4CV	It may be pulmonary veins pool. It is necessary to confirm PVs is connected with LA
Coronary sinus was visualized at the atrioventricular groove at the 4CV	It may be caused by the drainage of aberrant PVs or LSVC
3VT level	
A supernumerary Vein-like structure was visualized at the left side of the PA at the 3VT	It may be LSVC
A U-shaped confluence of ductal and aortic arches with the trachea and esophagus located between the two arches	Right-sided aortic arch; suggesting “rings and slings”

*3VT* three-vessel-trachea view, *4CV* four chamber view, *CHD* congenital heart disease, *DAO* descending aorta, *IVC*, inferior vena cava, *LA* left atrium, *LSVC* left superior vena cava, *PV* pulmonary vein, *RA* right atrium, *TAPVC* total anomalous pulmonary veins connection

Interobserver variability for Protocol A was determined by having another sonographer (Sonographer C) with similar experience re-analyze the imaging data in all the fetuses. For Protocol B, another sonographer (Sonographer D) who had undergone similar training program re-interpreted the imaging data to calculate the interobserver variability. Intraobserver variability for the two protocols were determined by having Sonographer C and D re-interpreted the imaging data in all the fetuses 30 days later under Protocol A and B, respectively. Interobserver and intraobserver variabilities for detecting CHDs were calculated as the absolute differences between the numbers of CHD cases detected by two sonographers as a percent of their mean.

### Statistical analysis

The detection rates for the two protocols were compared via McNemar analysis. *P*-values < 0.05 were considered statistically significant. All statistical analyses were performed using commercially available software (SPSS, release 17.0).

## Results

A total of 300 fetuses were included in the current study. All cases were confirmed by autopsy findings, operative findings, fetal/postnatal echocardiography, or by phone calls in order to acquire the information from routine infant physical examination reports. In our study, patent foramen ovale and patent ductus arteriosus were both considered normal cardiac structures during the follow-up.

In total, 52 cases of fetal CHD were included in the current study, of which five cases were from the routine screening pregnancies. Prenatal cardiac screenings for CHDs by the two protocols and fetal outcomes are summarized in Table 3. Sixteen families made a choice to terminate the pregnancy and four fetuses died within the uterus. For the living neonates, seven died and the other 25 survived the neonatal period. The detection rate for CHD under Protocol B was significantly higher than under Protocol A (96.2 % vs. 61.5 %, *P* < 0.01).

**Table 3:** Cardiac screening findings in confirmed CHD fetuses and clinical outcomes

Cardiac lesions (n)	Referred for echocardiography (n)		Outcomes (n)
	Protocol A	Protocol B	
Cardiac position			
Complete situs inversus (2)	0	2	NNA (2)
Dextrocardia (1)	1	1	TOP (1)
Heterotaxy (1)	0	1	NND (1)
Cardiomediastinal shift (2)	2	2	NNA (2)
Venous connection			
TAPVC (1)	0	1	NNA (1)
LSVC (2)	1	2	NNA (2)
LSVC without RSVC (1)	0	1	IUFD (1)
Interrupted IVC (1)	0	1	NNA (1)
Septal defects			
Large VSD (3)	3	3	NND (1); NNA (2)
Small VSD (2)	0	1	NNA (2)
CAVSD (2)	2	2	TOP (1); NNA (1)
PAVSD (1)	1	1	NNA (1)
Left heart anomalies			
HLHS (4)	4	4	TOP (4)
COA (1)	0	0	NND (1)
IAA and VSD (1)	1	1	TOP (1)
Right aortic arch (2)	0	2	NNA (2)
Aortic valve atresia (1)	1	1	IUFD (1)
Right heart anomalies			
HRHS (1)	1	1	TOP (1)
Ebstein’s anomaly (2)	2	2	IUFD (1); NNA (1)
Tricuspid dysplasia (1)	0	1	NND (1)
Tricuspid stenosis (1)	0	1	NNA (1)
Pulmonary atresia (2)	0	2	TOP (1); NND (1)
Pulmonary stenosis (1)	0	1	NNA (1)
Absent pulmonary valve (1)	1	1	IUFD (1)
Conotruncal anomalies			
TOF (2)	2	2	NNA (2)
TGA (3)	1	3	TOP (1); NND (2)
ccTGA (1)	0	1	NNA (1)
Taussig-Bing (1)	1	1	TOP (1)
DORV (3)	3	3	TOP (1); NNA (2)
CTA (2)	2	2	TOP (1); NNA (1)
Complex CHDs (3)	3	3	TOP (3)
Total (52)	32	50	NNA(25); NND (7); IUFD (4); TOP (16)

*CAVSD* complete atrioventricular septal defect, *ccTGA* congenitally corrected transposition of the great arteries, *COA* coarctation of aorta, *CTA* conotruncal anomalies, *DORV* double outlet right ventricle, *HLHS* hypoplastic left heart syndrome, *HRHS* hypoplastic right heart syndrome, *IAA* interrupted aortic arch, *IUFD* intrauterine fetal death, *IVC* inferior vena cava, *LSVC* left superior vena cava, *NNA*, neonatal alive, *NND* neonatal death, *PAVSD* partial atrioventricular septal defect, *RSVC* right superior vena cava, *TAPVC* total anomalous pulmonary venous connection, *TGA* transposition of the great arteries, *TOF* tetrology of Fallot, *TOP* termination of pregnancy, *VSD* ventricular septal defect

**Fig. 1:**
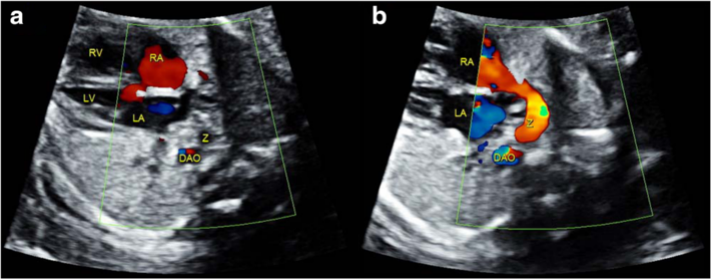
Sonographic images of a 22-gestational-week fetus diagnosed with interrupted IVC and azygos continuation. In the 4CV, a venous-like structure was detected adjacent to the DAO (**a**). Gradual rotation of the ultrasound beam; this structure was visualized to drain into the RA (**b**), and suspected to be the azygos. The abnormal structures were missed by sonographer under Protocol A, while they were detected under Protocol B as this sonographer knew that this sign was an indication of interrupted IVC and azygos continuation. 4CV, four-chamber view; DAO, descending aorta; LA, left atrium; LV, left ventricle; RA, right atrium; RV, right ventricle; Z, azygos

**Fig. 2:**
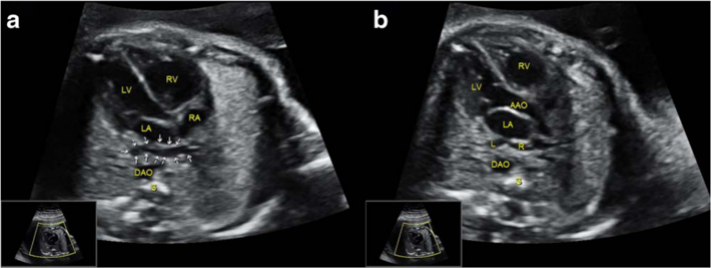
Sonographic images of a 22-gestational-week fetus diagnosed with TAPVC. In the 4CV, a tubular-like structure was detected between the LA and DAO (**a**). Gradual rotation of the ultrasound beam; two tiny structures were visualized to drain into the tubular-like structure (**b**). This was suspected to be TAPVC by screening sonographers under Protocol B, for whom this characteristic sign had been made evident. 4CV, four chamber view; AAO, ascending aorta; DAO, descending aorta; L, left; R, right; LA, left atrium; LV, left ventricle; RA, right atrium; RV, right ventricle; S, spine. The areas indicated by the *white arrows* depict the tubular-like structure

**Fig. 3:**
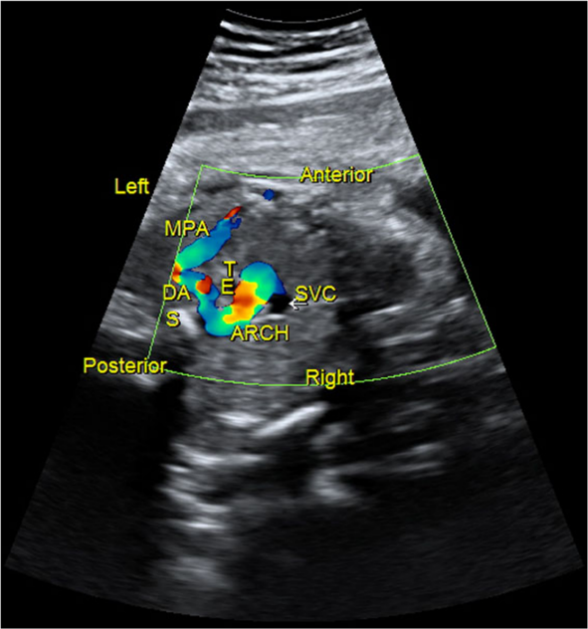
Sonographic images of a 23-gestational-week fetus diagnosed with right aortic arch. In the 3VT, the confluence of the DA and aortic arch formed a “U”-like structure, surrounding the trachea and esophagus. This was a characteristic sign of right aortic arch. 3VT, three-vessel-trachea view; DA, ductus arteriosus; E, esophagus; MPA, main pulmonary artery; S, spine; SVC, superior vena cava; T, trachea

**Fig. 4:**
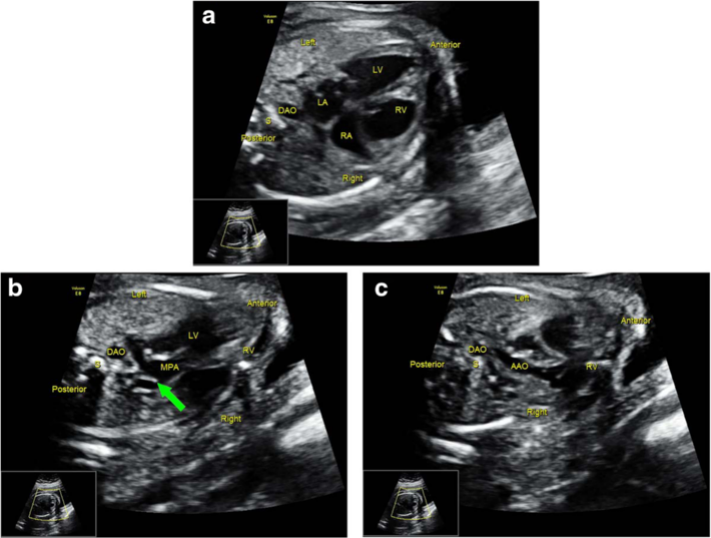
Sonographic images of a 22-gestational-week fetus diagnosed with TGA. The 4CV (**a**) shows four symmetric chambers with no obvious anomalies present. In the *left* (**b**) and *right* (**c**) outflow tract views, the connections of LV with MPA and RV with AAO are detected, respectively. Correct identification of the AAO and MPA was critical in making the diagnosis, which was accomplished by sonographers under Protocol B but failed under Protocol A. 4CV, four-chamber view; AAO, ascending aorta; DAO, descending aorta; LA, left atrium; LV, left ventricle; MPA, main pulmonary artery; RA, right atrium; RV, right ventricle; S, spine; TGA, transposition of the great arteries. The arrow indicates the bifurcation of the MPA

**Fig. 5:**
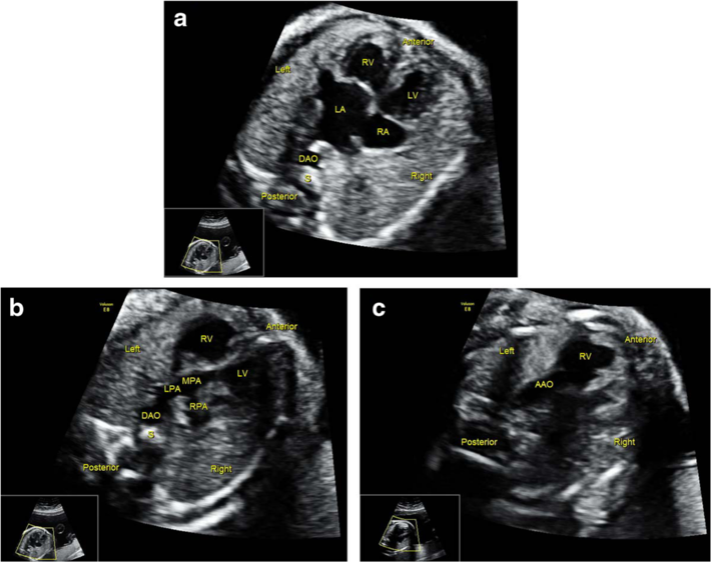
Sonographic images of a 22-gestational-week fetus diagnosed with ccTGA. The 4CV (**a**) showed four symmetric chambers that were considered to be normal by sonographers under Protocol A. However, it was confirmed as atrioventricular discordance by carefully visualizing the position of the atrioventricular valves that insert into the septum. In addition, the *left* (**b**) and *right* (**c**) outflow tract views substantiate that MPA was connected to morphological LV, while AAO connected to morphological RV. Sequential segmental analysis allowed sonographers under Protocol B to detect this anomaly. 4CV, four-chamber view; AAO, ascending aorta; ccTGA, congenitally corrected transposition of the great arteries; DAO, descending aorta; LA, left atrium; LPA, left pulmonary artery; LV, left ventricle; MPA, main pulmonary artery; RA, right atrium; RPA, right pulmonary artery; RV, right ventricle; S, spine

For fetuses with cardiac malpositions, only 50 % cases were detected under Protocol A, while all cases were visualized under Protocol B. We also found that sonographers easily detected malformations associated with systemic and pulmonary venous connections under Protocol B (100 %), while these anomalies were almost not noted under Protocol A. A movie file shows confirmation of situs in a case of heterotaxy in detail [See Additional file 1: Video]. Figure 1 and three additional movie files (See Additional files 2, 3 and 4: Video) show a case of suspected interrupted IVC and azygos continuation. Figure 2 and two additional movie files (See Additional files 5 and 6: Video) show a case of total anomalous pulmonary venous connection (TAPVC), in which the pulmonary veins were suspected to drain into the coronary sinus. Moreover, Protocol B was superior in detecting some lesions at the arterial level, such as right aortic arch (Fig. 3), transposition of the great arteries (TGA) (Fig. 4), congenitally corrected transposition of the great arteries (ccTGA) (Fig. 5), etc. Several additional movie files showed the detection of these cardiac anomalies in detail (See Additional file 7 for right aortic arch; Additional files 8, 9 and 10 for TGA; and Additional files 11, 12 and 13 for ccTGA: Video).

**Fig. 6:**
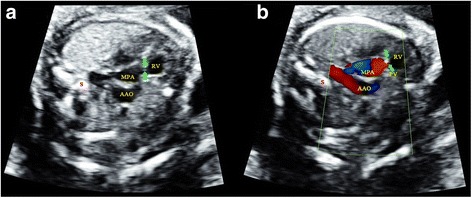
Sonographic images of a 24-gestational-week fetus diagnosed with pulmonary valve atresia. Thickened pulmonary valves (indicated by arrows) were visualized in the right outflow tract view (**a**) via 2D. However, this anomaly was neglected by the sonographers under Protocol A because the valvular movement was difficult to visualize at times. However, color Doppler resolved this problem by visualizing the retrograde flow in the MPA (**b**), indicating pulmonary valve atresia. At the same time, no blood flow was detected entering the MPA across the thickened PV. AAO, ascending aorta; MPA, main pulmonary artery; PV, pulmonary valve; RV, right ventricle; S, spine

**Fig. 7:**
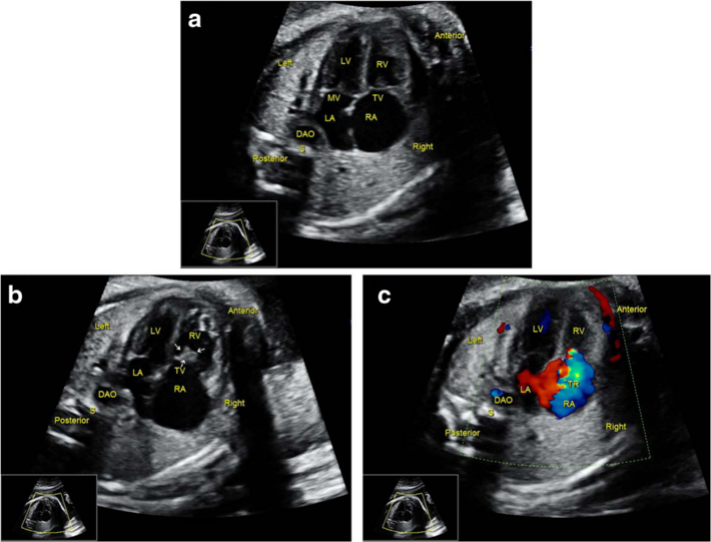
Sonographic images of a 24-gestational-week fetus diagnosed with tricuspid dysplasia. The 4CV was considered to be normal by sonographers under Protocol A because they did not find a thickened tricuspid valve (**a**). In fact, the RA was a little large in size according to careful observation. Also, the thickened tricuspid valves could be detected after careful scan (**b**). Color Doppler made it quite easy to detect tricuspid regurgitation, which would direct attention to valvular structural anomalies (**c**). DAO, descending aorta; LA, left atrium; LV, left ventricle; RA, right atrium; RV, right ventricle; S, spine; TR, tricuspid regurgitation; TV, tricuspid valve. The *arrows* indicate the thickened valves

Protocol B was efficient at detecting VSD, valvular stenosis/atresia/dysplasia, in which color Doppler might play an important role. Figure 6 and two additional movie files (See Additional files 14 and 15: Video) show a case of pulmonary valve atresia in which the lesion was barely visualized by 2D ultrasound as the PA was normal in diameter; but was easily detected by color Doppler due to the retrograde flow in DA and main PA. Figure 7 and two additional movie files (See Additional files 16 and 17: Video) show a case of tricuspid dysplasia with regurgitation, which was unobserved during routine screenings by 2D sonography. In fact, the thickened valves could only be visualized in certain views, while the regurgitation was very easily seen. For the CHD fetuses, the mean scanning and imaging interpretation times for cardiac screening were 8.7 ± 2.3 min and 17.2 ± 5.8 min for sonographers under Protocol A and B, respectively. Protocol B also required much more time than did Protocol A. For Protocol A, the interobserver and intraobserver variability was 6.5 and 9.8 % for detecting CHDs, respectively. The interobserver and intraobserver variability was 4.1 and 6.2 % for Protocol B, respectively.

Two hundred forty-eight fetuses with normal cardiac structures were also included in the current study. For these normal fetuses, the mean scanning and imaging interpretation times for cardiac screening were 5.6 ± 2.9 min and 8.3 ± 3.8 min for sonographers under Protocol A and B, respectively. Protocol B was again more time-consuming than Protocol A. Most of the normal fetuses were considered normal cardiac structures under both protocols. Table 4 summarizes the cardiac findings for those were suspected abnormal cardiac structures during screening but were confirmed to be normal by fetal/neonatal echocardiography.

**Table 4:** Cardiac screening findings in confirmed normal fetuses

Cardiac screening findings	Referred for further echocardiography (n)	
	Protocol A	Protocol B
Pericardial effusion	2	1
PAVSD	1	0
VSD	2	0
COA	1	1
Space-occupying lesions	1	1
Total	7	3

*COA* coarctation of aorta, *PAVSD* partial atrioventricular septal defect, *VSD* ventricular septal defect

**Fig. 8:**
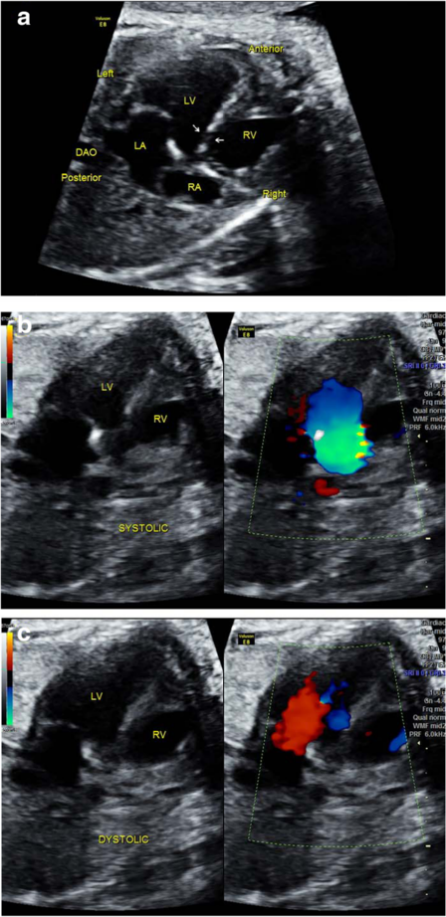
Sonographic images of a 24-gestational-week fetus in which VSD was falsely suspected. In the 4CV, a small echo drop-out was visualized at the perimembraneous septum, as indicated by the white arrows (**a**). This was at times easily considered to be a VSD, whereas it was actually an artifact. Color Doppler helped to resolve this problem. No flow signals were identified across the septum in either systolic (**b**) or diastolic (**c**) phases

**Fig. 9:**
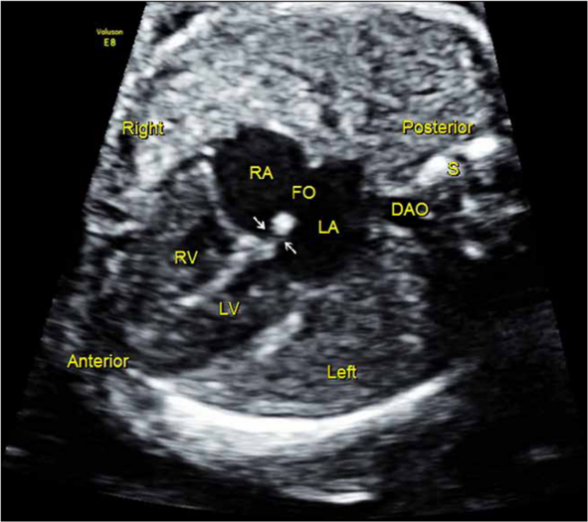
Sonographic images of a 21-gestational-week fetus in which PAVSD was falsely suspected. In the 4CV, a deficiency of the atrial septem primum was suspected by sonographers under Protocol A, as indicated by the white arrows. It was in actuality the echo of the coronary sinus which could be visualized when a non-standard 4CV was obtained with the section across the atrioventricular groove. These specific signs were mentioned in Protocol B

Some fetuses were referred for further echocardiography as pericardial effusion was suspected by sonographers under both Protocols A and B. In fact, this phenomenon has been mentioned in the ISUOG guidelines [16] as the small hypoechogenic rim around the fetal heart usually represents a normal variation. In one case, coarctation of the aorta (COA) was suspected mainly because of the angle of the ultrasound beam. In another case, a space-occupying lesion was suspected while in actuality it was the hypertrophic papillary muscle of the right ventricle. VSDs were also suspected falsely because of acoustic drop-out artifacts (Fig. 8). In another case, PAVSD was falsely suspected because the coronary sinus was regarded as a deficiency of the atrial septem primum (Fig. 9). It was interesting to note that Protocol B was better than Protocol A in reducing the false positives in detecting septal defects. Color Doppler also played a significant role in this phenomenon.

## Discussion

Of all the congenital anomalies, CHD is the most common and has the worst prognosis compared with other disorders due to its high morbidity and mortality [5, 6]. From the early 1990s, fetal echocardiography made it possible to detect CHDs prenatally [11, 12]. Initially, indications for fetal cardiac scans were only based upon some parental risk factors such as maternal diabetes or a positive family history of cardiac anomalies [22]. In the early 2000s, people realized that cardiac examinations should be extended to all fetuses undergoing routine obstetric scans [8, 23–25], and this challenged most obstetric sonographers as fetal cardiac scans and interpretations required a unique set of advanced expertise and knowledge.

Initially, obstetric sonographers recognized that 4CV was easy to visualize during routine scans, and that it was relatively easy to obtain the standard 4CV if the spinal acoustic shadow was avoided. The optimal view of the 4CV is usually obtained when the cardiac apex is directed toward the maternal wall. The introduction of 4CV in routine screenings has proven its value as it could detect some cardiac anomalies at the atrial and ventricular levels, such as large VSD, atrioventricular septal defect (AVSD), Ebstein’s anomaly, hypoplastic left heart syndrome (HLHS), hypoplastic right heart syndrome (HRHS) and space-occupying lesions, etc [9, 26]. In fact, it was relatively easy for us to visualize four asymmetric chambers during prenatal screenings, which provided evidence for further comprehensive examinations. Unfortunately, 4CV alone could not provide the anatomic information at the arterial level, which leads to the low detection rate for conotruncal anomalies [9, 27].

As more concerns have been raised about the low detection rate of 4CV screening, ISUGG revised their previously published guidelines for cardiac screening in mid-gestation. In the new version of the guidelines, transverse scans were recommended to involve both the 4CV and outflow tract views. Also, AIUM has proposed similar guidelines [28, 29]. It is therefore a very important step in improving detection of CHD by complementing the 4CV with the outflow tract views under these guidelines, as documented by several teams and regional studies [13, 15]. Theoretically, most cardiac anomalies can be detected if all the planes could be acquired and the image data interpretations are made correctly. However, our results were not as satisfactory as we had hoped. According to our study, the detection of CHD was only 61.5 % under the protocol using transverse scans. Though this detection rate might be underestimated, as we included more CHD fetuses in our study than in routine screenings, the results implicated the possibility during routine screenings of missed diagnoses for special CHDs. In our experience, we found many CHD neonates manifesting TAPVC, TGA, pulmonary atresia, etc., whose mothers had undergone prenatal screenings at our center but had received no positive fetal diagnosis. Lacking accurate prenatal diagnosis made it impossible to provide appropriate operative schemes and multidisciplinary care. Therefore, many of these neonates died and their families suffered great pain. In fact, as the largest prenatal screening and diagnosis center in northeast China, we have both the best ultrasound equipment and most experienced sonographers. We expect that there are possibly more CHDs misdiagnosed in rural areas, and this is why we decided to propose a new protocol to strengthen the overall efficiency of fetal cardiac screenings.

When the study was completed, we summarized the reasons for the low detection rate under the protocol using transverse scans (Protocol A). An echocardiographer could recognize and diagnose almost all of the CHDs in our study when carefully reviewing the recorded image data acquired from transverse scans. For example, in the case of TAPVC, the tubular-like echo between the descending aorta (DAO) and LA was an important clue to drawing attention to potential cardiac anomalies. However, sonographer under Protocol A did not visualize this abnormal structure because they were not aware that there were no special anatomical structures in the areas between LA and DAO. Also, in the case of left superior vena cava, no vessel-like structures should be detected at the left side of main pulmonary artery (MPA) for normal fetuses. Had the screening sonographers been made aware of this, they would not have made any mistakes. In our proposed new protocol (Protocol B), we solved this problem by introducing into our training program many ultrasonic characters that reflect special cardiac anomalies according to the pathologic anatomy. In fact, Jeanty et al. published a series of reviews [18–21] in which they summarized many of these useful ultrasonic characters for fetal CHDs, and some of these were referenced in our training program in Protocol B (mentioned in the Methods Section). The method proved its value in that the screening sonographers underwent relevant training and could then detect more CHDs than with other techniques.

Our newly proposed protocol emphasized confirming whether the connections of the two adjacent cardiac segments were correct or not. Sequential segmental analysis was recommended to evaluate the fetal cardiac structure and hemodynamcis. Although such analysis is a little difficult for screening sonographers, it justified its value by identifying all the TGA and ccTGA anomalies in the current study. As the 4CV appeared symmetric, these cardiac malformations were easily missed without a careful scan, even if the outflow tract views were scanned during routine screenings. On the contrary, identifying each anatomic structure at each segment, together with confirming the connections between each segment, ensured the successful detection of these complex anomalies.

In addition, the newly proposed protocol emphasized the application of color Doppler during cardiac scans and it justified its value by detecting more cardiac anomalies. For example, in the case of tricuspid dysplasia and tricuspid regurgitation, the thickened valves went undetected in the hands of a screening sonographer using only 2D, although they could be identified by an echocardiography specialist. In fact, color Doppler aids in the detection of flow disturbance, including stenosis and regurgitation, which may not be obvious from interrogation via 2D imaging alone. Furthermore, color Doppler discloses the flow direction in vessels, which is very useful in the detection of cardiac anomalies by assessing cardiac hemodynamics, and Jeanty et al [18–21] reported their experiences in their publications. In our study, we proved this technique’s value in the case of pulmonary atresia in detecting retrograde flow in MPA and DA. As the valvular movement was difficult to observe via 2D, detection of this cardiac abnormality was missed by sonographer under Protocol A, in which color Doppler application was not mandatory.

We must state that there are some limitations to our proposed protocol. First, systematic training was needed to carry out this protocol, which was expensive and time-consuming. However, a 6-month training program has proven to be of great value in improving the efficiency of detecting CHDs during screening. Government investment and funding support may solve this problem. In addition, scans and imaging interpretations under the new protocol, based on sequential segmental analysis, require more time and expertise than under the previous screening protocol. However, we believe that the time commitment might be reduced gradually with the increase in proficiency. In our hands, the time spent scanning and interpreting images was about 15 min for a fetus with normal cardiac structures under the new protocol when the program began, which was then reduced to about 5 to 10 min by the time the study was terminated.

The current study was limited in that it was a relatively small study and did not represent a normal population, i.e., more CHD fetuses were intentionally entered into the study, not reflecting a non-selected population that underwent prenatal screenings. This overall design may bias the results so as to overestimate the difference between the two protocols. We emphasized to summarize the reasons for the cases being missed or detected by the two protocols, as the overall aim of the study was to improve cardiac screening efficiency by proposing a new protocol. As a mono-center based, short-term study, it might be impossible to observe various kinds of CHDs during routine screenings, and the spectrum of CHDs might vary greatly. The current design, then, was intended to include more CHD fetuses to evaluate the ability of the two protocols in screening CHDs, which might compromise the study. However, these limitations, had they been addressed in a multi-center study in a normal screening population, would likely have led to improvements in the completeness of the studies, strengthening our conclusion that the newly proposed protocol was more efficient in detecting CHDs.

The study was also limited in that all fetuses were scanned by only one sonographer under each protocol. The design limited the scanning time for fetal ultrasound examinations under the principle of exposure “as low as reasonably achievable.” Alternatively, we made inter- and intro- variability analysis by having another two sonographers with similar experience to re-analyze the imaging data and made interpretations, respectively. However, this may bias the results as one sonographer only read the videos recorded by the other. If the videos were of poor quality (i.e., a non-standard view, or critical information not presented well), the detection of CHD by the sonographer who did not perform the scan was inevitably low and underestimated.

## Conclusions

The current study introduces an enhanced protocol for fetal cardiac screening, under which the obstetric screening sonographers identify the fetal cardiac anatomy and hemodynamics systematically. Some specific ultrasonic signs in certain views help to screen for specific cardiac anomalies. A short-term training program makes it possible for the screening sonographers to become familiar with the new protocol; this has confirmed its value in improving screening efficiency.

## Abbreviations

2D, two-dimensional; 3VT, three-vessel and trachea view; 4CV, four-chamber view; AVSD, atrioventricular septal defect; ccTGA, congenitally corrected transposition of the great arteries; CHD, congenital heart disease; COA, coarctation of the aorta; DA, ductus arteriosus; DAO, descending aorta; HLHS, hypoplastic left heart syndrome; HRHS, hypoplastic right heart syndrome; IVC, inferior vena cava; LA, left atrium; LV, left ventricle; MPA, main pulmonary artery; PA, pulmonary artery; PAVSD, partial atrioventricular septal defect; PV, pulmonary vein; RA, right atrium; RV, right ventricle; SVC, superior vena cava; TAPVC, total anomalous pulmonary vein connection; TGA, transposition of the great arteries; VSD, ventricular septal defect

## Additional files

Additional file 1: Video. Identification of fetal situs in the case of a fetus at 23 gestational weeks that was diagnosed with heterotaxy. The first step was to determine fetal head position and posture. The ultrasound beam was then rotated 90° counterclockwise from the initial position in order to see the transverse plane of the fetus. We swept gradually to observe the transverse plane at both the 4CV and up-abdominal levels. The spine could be used as the landmark to identify the left or right side of the fetus. In this case, the heart was on the left side while the gastric vacuole was on the right side. (AVI 3734 kb) Additional file 2: Video. Identification of an interrupted inferior vena cava and azygos continuation in the case of a fetus at 22 gestational weeks. In the four-chamber view, a venous-like structure was visualized beside the aorta, which was confirmed to drain into the right atrium when we gradually rotated the ultrasound beam. (AVI 3152 kb) Additional file 3: Video. Identification of an interrupted inferior vena cava and azygos continuation in the case of a fetus at 22 gestational weeks. In the four-chamber view, a venous-like structure was visualized beside the aorta, which was confirmed to drain into the right atrium when we gradually rotated the ultrasound beam. (AVI 2477 kb) Additional file 4: Video. Identification of an interrupted inferior vena cava and azygos continuation in the case of a fetus at 22 gestational weeks. No inferior vena cava was detected draining into the right atrium directly. (AVI 2477 kb) Additional file 5: Video. Visualization of a total anomalous pulmonary vein connection in the case of a fetus at 22 gestational weeks. In the four-chamber view, an abnormal tubular-like structure was present between the area of the left atrium and the descending aorta, which was confirmed to drain into the coronary sinus and ultimately into the right atrium. Thus, this case proved to be a total anomalous pulmonary vein connection. (AVI 2927 kb) Additional file 6: Video. Visualization of a total anomalous pulmonary vein connection in the case of a fetus at 22 gestational weeks. In the four-chamber view, an abnormal tubular-like structure was present between the area of the left atrium and the descending aorta, which was confirmed to drain into the coronary sinus and ultimately into the right atrium. Thus, this case proved to be a total anomalous pulmonary vein connection. (AVI 4277 kb) Additional file 7: Video. Identification of the right aortic arch in the case of a fetus at 23 gestational weeks. At the three-vessel and tracheal view, the confluence of the ductus arteriosus and aortic arch formed a “U”-like structure, surrounding the trachea and esophagus. It was a characteristic sign for the right aortic arch. (AVI 4727 kb) Additional file 8: Video. Identification of transposition of the great arteries in the case of a fetus at 22 gestational weeks. The four-chamber view showed no obvious abnormalities. (AVI 4277 kb) Additional file 9: Video. Identification of transposition of the great arteries in the case of a fetus at 22 gestational weeks. The outflow tract view demonstrated that the pulmonary artery was connected with the left ventricle. (AVI 5853 kb) Additional file 10: Video. Identification of transposition of the great arteries in the case of a fetus at 22 gestational weeks. The outflow tract view demonstrated that the aorta was connected with the right ventricle. (AVI 3602 kb) Additional file 11: Video. Identification of congenitally corrected transposition of the great arteries in the case of a fetus at 22 gestational weeks. The four-chamber view showed a symmetric four-chambered heart, while atrioventricular discordance was demonstrated after careful analysis. The left atrium was connected with the morphologic right ventricle; the right atrium connected with the morphologic left ventricle. (AVI 3152 kb) Additional file 12: Video. Identification of congenitally corrected transposition of the great arteries in the case of a fetus at 22 gestational weeks. In the outflow tract view, ventriculoarterial discordance was identified: the pulmonary artery was connected with the morphologic left ventricle. (AVI 5853 kb) Additional file 13: Video. Identification of congenitally corrected transposition of the great arteries in the case of a fetus at 22 gestational weeks. In the outflow tract view, ventriculoarterial discordance was identified: the right ventricle was connected with aorta. (AVI 8553 kb) Additional file 14: Video. Identification of pulmonary valve atresia in the case of a fetus at 24 gestational weeks. Thickened pulmonary valves with restrictive openings could be detected upon careful scan. (AVI 5403 kb) Additional file 15: Video. Identification of pulmonary valve atresia in the case of a fetus at 24 gestational weeks. Color Doppler made it easy to diagnose as no flow was visualized entering the pulmonary artery across the pulmonary valves. At the same time, retrograde flow could be detected in the main pulmonary artery, which was an indirect sign of pulmonary valve atresia. (AVI 5178 kb) Additional file 16: Video. Identification of tricuspid dysplasia in the case of a fetus at 24 gestational weeks. The opening of the tricuspid valve appeared normal at times by 2D sonography, while color Doppler showed apparent tricuspid regurgitation. The thickened tricuspid valves were detected by detailed rescan. (AVI 3827 kb) Additional file 17: Video. Identification of tricuspid dysplasia in the case of a fetus at 24 gestational weeks. The opening of the tricuspid valve appeared normal at times by 2D sonography, while color Doppler showed apparent tricuspid regurgitation. The thickened tricuspid valves were detected by detailed rescan. (AVI 3827 kb)
